# Evaluation of Self-Management Support Functions in Apps for People With Persistent Pain: Systematic Review

**DOI:** 10.2196/13080

**Published:** 2019-02-12

**Authors:** Hemakumar Devan, Devin Farmery, Lucy Peebles, Rebecca Grainger

**Affiliations:** 1 Centre for Health, Activity and Rehabilitation Research, School of Physiotherapy University of Otago Wellington New Zealand; 2 Department of Medicine, University of Otago Wellington New Zealand; 3 University of Otago Wellington New Zealand

**Keywords:** smartphone, chronic pain, culture, mHealth, self-management, technology

## Abstract

**Background:**

Smartphone apps are a potential mechanism for development of self-management skills in people with persistent pain. However, the inclusion of best-practice content items in available pain management apps fostering core self-management skills for self-management support is not known.

**Objective:**

The aim of the study was to evaluate the contents of smartphone apps providing information on pain management strategies for people with persistent pain facilitating self-management support and to appraise the app quality.

**Methods:**

A systematic search was performed in the New Zealand App Store and Google Play Store. Apps were included if they were designed for people with persistent pain, provided information on pain self-management strategies, and were available in English. App contents were evaluated using an a priori 14-item self-management support (SMS-14) checklist. App quality was assessed using the 23-item Mobile Apps Rating Scale.

**Results:**

Of the 939 apps screened, 19 apps met the inclusion criteria. Meditation and guided relaxation were the most frequently included self-management strategies. Overall, the included apps met a median of 4 (range 1-8) of the SMS-14 checklist. A total of 3 apps (Curable, PainScale-Pain Diary and Coach, and SuperBetter) met the largest number of items (8 out of 14) to foster self-management of pain. Self-monitoring of symptoms (n=11) and self-tailoring of strategies (n=9) were frequently featured functions, whereas a few apps had features facilitating social support and enabling communicating with clinicians. No apps provided information tailored to the cultural needs of the user. The app quality mean scores using Mobile Apps Rating Scale ranged from 2.7 to 4.5 (out of 5.0). Although use of 2 apps (Headspace and SuperBetter) has been shown to improve health outcomes, none of the included apps have been evaluated in people with persistent pain.

**Conclusions:**

Of the 3 apps (Curable, PainScale-Pain Diary and Coach, and SuperBetter) that met the largest number of items to support skills in self-management of pain, 2 apps (PainScale-Pain Diary and Coach and SuperBetter) were free, suggesting the potential for using apps as a scalable, wide-reaching intervention to complement face-to-face care. However, none provided culturally tailored information. Although 2 apps (Headspace and SuperBetter) were validated to show improved health outcomes, none were tested in people with persistent pain. Both users and clinicians should be aware of such limitations and make informed choices in using or recommending apps as a self-management tool. For better integration of apps in clinical practice, concerted efforts are required among app developers, clinicians, and people with persistent pain in developing apps and evaluating for clinical efficacy.

## Introduction

### Background

Persistent, noncancer pain is the leading cause of disability worldwide, affecting 1 in 5 people [1,2]. Persistent pain includes a wide range of pain conditions such as persistent primary pain (eg, low back pain and neck pain), musculoskeletal pain (eg, osteoarthritis and rheumatoid arthritis), migraine, orofacial pain, neuropathic pain, and pain following trauma and surgery [3]. Recent results from the Global Burden of Diseases suggest persistent low back pain and migraine are the 2 major contributors globally to disability-adjusted life years [4], signifying the large personal, societal, and economic impact of persistent pain.

The current best practice for management of persistent pain involves group-based, multidisciplinary, cognitive behavioral interventions focusing on fostering self-management [5-7]. Self-management support can be achieved by providing individuals with tools and strategies to help them choose healthier behaviors and transforming the patient-clinician relationship into one of collaborative care [8]. Fostering self-management support could be achieved via core self-management skills. Core self-management skills include self-efficacy building, self-tailoring, self-monitoring of symptoms, goal setting and planning, problem solving, and shared decision making. In the context of persistent pain, individuals learn a variety of self-management or active coping strategies (eg, cognitive behavioral therapy, activity pacing, and relaxation techniques) from pain management interventions. In collaboration with health professionals (*shared decision making*), individuals learn to identify meaningful goals (eg, personal, physical, and psychological) to improve pain and functioning (*goal setting*) [8]. Practicing a variety of strategies learned from pain management interventions, individuals develop the capacity to monitor their symptoms (*self-monitoring*) and tailor strategies that work best for them (*self-tailoring*) [5]. With the help and support of peers and health professionals, they can actively problem solve (*problem solving*) and set meaningful goals [9]. Positive reinforcement from learning self-management skills and adhering to practicing those strategies [10] ultimately develops the confidence in one’s ability (*self-efficacy building*) to engage in meaningful activities despite pain (*acceptance*), thereby fostering sustained behavioral change [11].

Specialized pain services including multidisciplinary, cognitive behavioral interventions are resource intensive and are often only provided in secondary or tertiary care settings [5]. Therefore, there are many barriers to accessing specialized pain services in Australia and New Zealand, including long-waiting lists after referral from primary care [12], issues related to transport, and physically accessing in-person delivered pain services [13]. Furthermore, there is little capacity to increase services because of a lack of a specialist pain workforce [12]. Therefore, innovative solutions for delivering pain management services are urgently required.

Smartphone apps are a potential mechanism for the development of self-management skills. Interventions delivered via smartphones are more likely to be easily accessible to a wider population and are scalable [14-16], with smartphone user numbers expected to reach 5 billion in 2020 [17]. Evidence suggests smartphone app use can foster self-management skills in other long-term conditions such as diabetes, asthma, and gout [18-20], and it can potentially deliver behavioral interventions to people living with persistent pain [21,22]. For example, pain apps have been generally classified to (1) provide general information on pain, including symptom identification and planning treatments, (2) track daily symptoms such as pain intensity, mood, daily activity, and medications, and (3) provide information on self-management strategies [23].

Previous reviews have examined the quality and content of self-management apps for people with persistent pain [23,24]. However, none of these reviews evaluated the app contents using a comprehensive self-management support checklist [3], including the cultural appropriateness of the contents via addressing cultural beliefs (eg, race, ethnicity, religion, and socioeconomic status). Cultural beliefs have been shown to influence illness perceptions, pain experiences, and attitudes to pain management [25-27], and addressing cultural beliefs of individuals with persistent pain is recommended by international clinical guidelines for pain management [28,29]. Lalloo et al [24] comprehensively reviewed 256 pain management apps in 2014 and classified the contents based on 5 core self-management skills (pain education, self-efficacy building, self-monitoring, social support, and goal setting). The review identified the lack of a comprehensive app, including all the self-management skills and lack of health care provider involvement in over 91.7% (256/279) of the included apps. However, the included apps were not described in detail, and there was no quality evaluation of the apps. Recently, reviews of self-management apps for specific pain conditions, namely persistent low back pain and arthritis, have been performed [30,31]. The included apps in both reviews recommended evidence-based interventions, yet the quality of information in the apps was poor [30,31]. Although Machado et al [30] concluded that there remains a need for higher-quality apps fostering self-management in people with persistent low back pain, Bhattarai et al [31] suggested the need for apps better suited for older adults, the more prevalent population group with arthritis. However, the apps identified from both reviews are not suitable for people with a wide variety of persistent pain conditions.

### Objectives

In light of such limitations, this review extends previous work by adopting a comprehensive International Classification of Diseases (ICD-11) definition for persistent pain [3] and evaluating app contents using an evaluation checklist for best-practice content items for self-management support. The primary purpose of the review was to evaluate the contents of smartphone apps providing information on pain management strategies for people with persistent pain facilitating self-management support and to appraise app quality.

## Methods

This review was reported according to the Preferred Reporting Items for Systematic Reviews and Meta-Analyses guidelines for systematic reviews [32]. The review protocol was submitted for registration at PROSPERO—International Prospective Register of Systematic Review; however, the submission was rejected as PROSPERO does not accept systematic review protocols for apps.

### Operational Definition

Persistent pain was defined as recurrent pain for more than 3 months [3], and persistent pain disorders were classified based on the ICD-11 definition [3] as (1) persistent primary pain, (2) persistent posttraumatic and postsurgical pain, (3) persistent neuropathic pain, (4) persistent malignant pain, (5) persistent headache and orofacial pain, (6) persistent visceral pain, and (7) persistent musculoskeletal pain. Culture was defined as a broad construct encompassing ethnicity, religion, socioeconomic status, disability, and sexual orientation [33].

### Search Strategy

A systematic search was performed in New Zealand Google Play Store (Android) and the App Store (iOS)—via the website fnd.io [34] —on November 14, 2017. These stores account for 99.22% of the market share of smartphone platforms used in New Zealand [35]. Search terms for both stores included *pain*, *pain management*, and *chronic pain*. An updated search was conducted on December 07, 2018, using the term *pain management* in both Google Play and App stores (via fnd.io [34]) to identify any new apps.

### Inclusion Criteria

Apps were included if they were (1) smartphone apps targeted to adults (>16 years) with persistent pain, (2) capable of being used for any persistent pain condition, (3) able to provide information on at least one or more of the following recommended strategies for pain management: pain education, activity pacing, thought and behavioral management, exercises, relaxation or breathing, meditation or mindfulness, distraction techniques, and (4) available in English.

Apps were excluded if (1) a specific pain condition was targeted (eg, low back pain and migraine), (2) only pain monitoring function was provided, and (3) the app was an advertisement for a specific clinic or product.

### Data Extraction

App names and descriptions from the app search in Google Play Store and the App Store were screened against a priori selection criteria. Next, eligible apps were downloaded for further screening using either OnePlus 3 smartphone running Android 8.0.0 for Google Play Store apps or Apple iPhone 6S running iOS 11.1.2 for the iOS platform. Apps found on both platforms were only downloaded on the iOS platform [30]. DF conducted the app search and screened apps for inclusion. Unclear app store descriptions were discussed with HD or RG for selection. When a free version (often indicated by *lite* in the title) and fully featured paid version of a same app were available, we assessed only the fully featured paid version so that the full functionality of the app was evaluated. A list of the final included apps was collated into an Excel spreadsheet (Microsoft Corp), with metadata about each app extracted from the relevant app stores. This included information on developer, price, app size (in megabytes), app version, and a brief summary of app contents.

### Evaluation of App Contents for Self-Management Support

Currently, there are no widely accepted frameworks or clinical guidelines recommending the key components of a pain self-management program. Therefore, a customized 14-item self-management support (SMS-14) checklist [5,8,24,36] was used for the purpose of this app review to evaluate the contents of the included apps for learning or developing self-management support (Table 1). The SMS-14 checklist included 6 core self-management skills (12 items) and 2 functions (2 items). Core self-management skills included self-efficacy building via recommended pain management strategies (7 items), problem-solving (1 item), goal-setting (1 item), self-tailoring (1 item), self-monitoring (1 item), and partnership between views of the patient and clinician via communication skills (1 item). The functions included providing access to social support (1 item) and culturally tailored information (1 item) by addressing cultural beliefs related to ethnicity, religion, socioeconomic status, disability, and sexual orientation [33]. The items from the SMS-14 checklist were informed from a well-established self-management framework—Stanford Self-management Support model [8,36], an evidence-based persistent pain guideline [28]—and previous reviews on self-management and persistent pain [5,24,31]. Apps scored 1 point for each item featured within the app, with a possible score between 0 and 14. Two reviewers (DF and HD) independently evaluated the SMS-14 checklist of included apps by using all the functions of the downloaded apps for at least 10 min and scored the items based on mutual consensus. RG reviewed and adjudicated any discrepancies.

### App Quality Evaluation

The 23-item Mobile Apps Rating Scale (MARS) [37] was used to assess general app quality. The MARS is a reliable tool for assessing app quality using 5 sections: engagement (5 items), functionality (4 items), aesthetics (3 items), information quality (7 items), and a subjective app quality score (4 items). The app quality mean score of an included app was calculated from individual mean scores of engagement, functionality, esthetics, and information quality. Two trained reviewers (LP and DF) independently scored the included apps using the MARS and subsequently compared scores and formed a consensus opinion to give a final reported MARS score [37]. Any difficulties in reaching a consensus were discussed with HD to reach a conclusion. For each MARS section, the interrater reliability of total mean scores between the reviewers was calculated using intraclass correlation coefficient (ICC)—2-way random-effects model with absolute agreement among single ratings [1,3].

**Table 1:** Self-management support (SMS-14) checklist for best-practice content items for self-management support of pain.

Skills	Description	Examples (if one or more present, scored *yes*)	Score
Self-efficacy building	Provision of information on self-management or active coping strategies to improve the ability to control one’s behavior—Cognitive Behavioral Therapy (CBT) approaches	Pain education: Mechanisms of pain/neurophysiology; Information on pain-stress-depression; Thought management; Fear avoidance; Catastrophizing; Medication use; Sleep management	Yes; No
		Activity pacing: Increments in activity interspersed with short periods of rest or changes in activity not determined by level of pain; (eg, increasing tolerance on activities of preference such as walking and sitting)	Yes; No
		Thought and behavioral management: CBT-based therapy; Acceptance-based therapies: Mindfulness-based stress reduction; Acceptance and commitment therapy	Yes; No
		Exercises (biomechanical or aerobic): Biomechanical—Exercises aimed at changing or improving spinal mechanics (eg, stretching, strengthening, range of motion, Pilates, and McKenzie exercises); Aerobic exercises—Exercises aimed at improving cardiovascular fitness and endurance (eg, walking, running, and swimming)	Yes; No
		Relaxation and breathing: Self-calming methods—Breathing exercises and mental imagery	Yes; No
		Meditation and mindfulness: Interventions primarily focusing on physical, mental, and spiritual focus; Yoga, Tai Chi, and mindfulness.	Yes; No
		Distraction techniques: Shifting attentional focus from pain; Music, art, play, and images	Yes; No
Self-tailoring	Provision of structured information and self-management support based on the individual symptoms/needs	Scope for the individuals to incorporate the self-management strategies learnt to fit their individual needs	Yes; No
Self-monitoring of symptoms	Capacity to help people to monitor their symptoms (eg, mood, thoughts, and pain intensity)	Thought diaries; Daily activity tracking; Pain diaries; Mindfulness; Sleep management	Yes; No
Goal setting and planning	Capacity to identify and log meaningful goals (physical, emotional, social) and track goals	Planning daily activities; Planning a specific activity goal	Yes; No
Problem solving	A systematic approach to be aware of and developing a plan for dealing with stressful or challenging situations	Having a plan for dealing with flare-ups; Ability to deal with stressors (eg, pain, stress, depression, and anxiety)	Yes; No
Partnership between views of patient and clinicians	Opportunity to interact with health care provider and involve people with persistent pain in decision making	Information/training on assertive communication with health professionals for patients	Yes; No
Social support	Access to a community of persons living with persistent pain	Provision of access to other persons in pain through the app for the purpose of providing emotional, informational, and appraisal support	Yes; No
Cultural relevance	Reporting of culturally tailored information applicable for diverse ethnic groups	Tailored information toward different cultures: Ethnicity; Religion; Socioeconomic status; Disability; Sexual orientation	Yes; No

## Results

### Systematic Search

**Figure 1:**
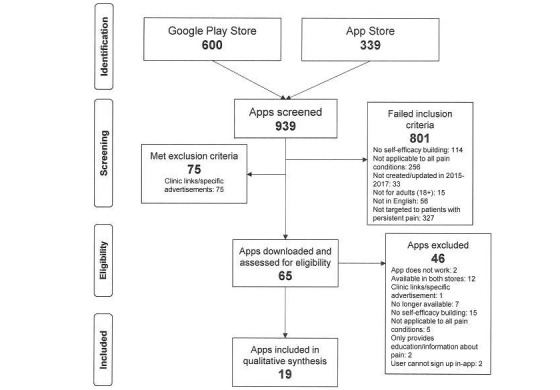
Flowchart for systematic app search from Google Play and App stores.

The initial search identified 600 unique Android apps from the Google Play Store and 339 unique iOS apps from the App Store—Figure 1. Following app name and description screening, 876 apps were excluded. Reasons for app exclusion were not being relevant to people with persistent pain (327/876, 37.3%), not applicable to all pain conditions (256/876, 29.2%), and not containing information related to pain management strategies (114/876, 13.0%)—Figure 1. After full app content screening, 19 apps were included for the review. This included 1 app (Curable) found only in the updated search.

### App Characteristics and Content

A summary of key characteristics of included apps is presented in Table 2. Overall, 12 of the 19 included apps were on both platforms, 3 were only on Android, and 4 only on iOS. Apps ranged in size from 3.3 MB to 105.30 MB, with the largest apps being Mindfulness Daily (105.3 MB) and Release Pain with Andrew Johnson (97.3 MB). Moreover, 5 of the apps were free and 11 had a single payment ranging from NZD $2.99 to NZD $14.99. Additionally, 3 apps were available on annual subscription (Aware—Meditation and Mindfulness NZD $49.99, Curable NZD $209.88, and Headspace NZD $139.99), and some of these apps also required additional in-app downloads for specific pain management content. Just more than half of the included apps (n=10) explicitly described the involvement of a health professional in developing app contents. A majority of the included apps were meditation (n=13) and relaxation apps (n=16) in the form of audio-guided imagery and hypnosis. One app provided comprehensive pain neuroscience education and guided relaxation for pain management (Curable). In addition, 2 apps delivered yoga in the form of postures for pain relief (Yoga for Pain therapy) and a specific form of transcendental meditation (Sahaja Kundalini Meditation).

Overall, the 19 apps met a median of 4 (range 1-8) of the SMS-14 checklist (Table 3). Free apps (median=7, range 2-8) scored better than the paid apps (median=1.5, range 1-8), with 2 of the 3 highest scoring apps (Pain Scale—Pain Diary and Coach and SuperBetter both scored 8) being free. Of the paid apps, the Curable app had the top score, including 8 content items from the SMS-14 checklist. Self-tailoring of provided skills and strategies (n=9) and the capacity for self-monitoring of symptoms (diary entries and/or self-reflection) (n=11) were both commonly found self-management skill content. Apps featuring cognitive interventions such as meditation (n=13) scored well for self-management score, as this content often gained 1 point for each of relaxation and breathing, thought and behavioral management, and problem solving. Of the 7 self-efficacy building strategies, pain education, activity pacing, exercises, and distraction techniques were seldom included. Opportunities to communicate with clinicians, social support from peers with persistent pain, and features to actively set goals and problem solve their symptoms were found infrequently, and none of the apps contained tailored information to address cultural beliefs. The mutual agreement between DF and HD’s independent evaluations of SMS-14 checklist was substantial; adjusted kappa statistic (κ) was 0.86 (95% CI 0.79-0.93).

### Quality Assessment

The app quality mean scores from MARS evaluation ranged from 2.72 to 4.54 (Table 4). Curable (4.54), Headspace (4.48), and PainScale (4.47) were the 3 highest scoring apps. Out of the 5 sections of the MARS, apps scored poorly in engagement and subjective app quality. Overall, 4 apps met criterion for scientific validation (item 19; Relax—Stress and Anxiety Relief, Mindfulness Daily, Headspace, and SuperBetter), of which 2 (Headspace and SuperBetter) had been clinically evaluated to show improved health outcomes [38,39]. No apps scored for scientific validation in improving health outcomes for people with persistent pain. The ICC scores for all sections from our MARS rating were greater than 0.69; in particular, app quality mean score showed a high level of reliability (ICC=0.92), indicating a good consistency across app ratings.

**Table 2:** Summary of app characteristics.

App Name	Developer	Platform(s)	App Version	Cost (NZD)	App size (MB)	Description
Aware—Meditation and Mindfulness	Uber Health Tech Pvt. Ltd	Android or iOS	2.2	$49.99 per year RRP^a^	8.19	Mindfulness or guided meditation
Counting Down to Relaxation	Healthy Visions	Android or iOS	2.1	$4.49	24.38	Guided relaxation or hypnosis
Curable: Back Pain, Migraine and Chronic Pain Relief	Curable Inc	Android or iOS	4.1.0	$209.88 per year RRP^a^	3.44	Guided mind and body strategies for pain management
Freedom From Pain	Healthy Visions	Android or iOS	2.1	$12.99	24.85	Hypnosis audio track
General Pain Management Guided Imagery	Shelley Spencer-Hellmich	Android or iOS	2	$8.99	48.3	Guided relaxation audio
Headspace: Guided Meditation	Headspace, Inc.	Android or iOS	3.2.4	$139.99 per year RRP^a^	30.28	Guided mindfulness to increase *approach* attention
Meditations for Pain Relief	Highly Meditated	Android	1.0.0	$3.02	37.54	Mindfulness or guided meditation
Mindfulness Coach	US Department of Veterans Affairs (VA)	iOS	1.4	Free	24.8	Mindfulness or guided meditation
Mindfulness Daily	INWARD, INC	iOS	1.4	Free	105.3	Mindfulness or guided meditation
Mindfulness Meditation for Pain Relief—Jon Kabat-Zinn	Sounds True	iOS	101.101.5	$14.99	32.1	Mindfulness or guided meditation
Pain Control Hypnosis by Glenn Harrold	Diviniti Publishing Ltd	Android or iOS	1.3	$5.99	80.54	Hypnosis audio
Pain Relief and Healing Meditation	Drentek	Android or iOS	2	$2.99	51	Mindfulness or guided meditation
Pain Relief Hypnosis PRO	Surf City Apps LLC	Android or iOS	4.5	$5.99	68.3	Hypnosis audio
PainScale-Pain Diary and Coach	Boston Scientific	Android or iOS	1.5	Free	31.7	Pain monitoring and management
Relax—Stress and Anxiety Relief	Saagara	Android or iOS	2.03	$4.49	51	Guided relaxation audio
Release Pain with Andrew Johnson	Michael Schneider	iOS	8.38	$4.49	97.3	Guided relaxation audio
Sahaja Kundalini Meditation	egeApps	Android	1.2.6	$4.49	7.56	Guided Kundalini meditation
SuperBetter	SuperBetter, LLC	Android or iOS	1.1.4	Free	4.91	Gamified tasks and goal-setting
Yoga For Pain Therapy	Harwell Publishing	Android	2	Free	16.02	Yoga postures and meditation

^a^RRP: recommended retail price.

**Table 3:** Evaluation checklist for self-management skills and functions.

App name	Core self-management skills												Functions		Total (14)
	Self-efficacy building							ST^h^	SM^i^	GS^j^	PS^k^	PV^l^	SS^m^	CR^n^	
	PE^a^	AP^b^	TB^c^	E^d^	R/B^e^	M/M^f^	D^g^								
Aware—Meditation and Mindfulness	—^o^	—	✓	—	✓	✓	—	✓	✓	—	✓	—	—	—	6
Counting Down to Relaxation	—	—	—	—	✓	—	—	—	—	—	—	—	—	—	1
Curable: Back Pain, Migraine and Chronic Pain Relief	✓	—	✓	—	✓	✓		✓	✓	—	✓	—	✓	—	8
Freedom From Pain	—	—	—	—	✓	—	—	—	—	—	—	—	—	—	1
General Pain Management Guided Imagery	—	—	—	—	✓	—	—	—	—	—	—	—	—	—	1
Headspace: Guided Meditation	—	—	✓	—	✓	✓	—	✓	✓	—	✓	—	—	—	6
Meditations for Pain Relief	—	—	—	—	✓	✓	—	—	✓	—	✓	—	—	—	4
Mindfulness Coach^p^	—	—	✓	—	✓	✓	—	✓	✓	✓	—	—	—	—	6
Mindfulness Daily^p^	—	—	✓	—	✓	✓	—	✓	✓	✓	✓	—	—	—	7
Mindfulness Meditation for Pain Relief—Jon Kabat-Zinn	—	—	✓	—	✓	✓	—	✓	✓	—	✓	—	—	—	6
Pain Control Hypnosis by Glenn Harrold	—	—	—	—	✓	—	—	—	—	—	—	—	—	—	1
Pain Relief & Healing Meditation	—	—	—	—	✓	✓	—	—	—	—	—	—	—	—	2
Pain Relief Hypnosis PRO	—	—	—	—	✓	—	—	—	—	—	—	—	—	—	1
PainScale-Pain Diary and Coach^p^	✓	—	—	✓	✓	✓	—	✓	✓	—	—	✓	✓	—	8
Relax—Stress and Anxiety Relief	—	—	—	—	✓	✓	—	✓	✓	—	—	—	—	—	4
Release Pain with Andrew Johnson	—	—	—	—	✓	—	—	—	—	—	—	—	—		1
Sahaja Kundalini Meditation	—	—	—	—	—	✓	—	—	—	—	—	—	—	—	1
SuperBetter^p^	—	✓	✓	✓	—	✓	—	✓	✓	✓	✓	—	✓	—	8
Yoga For Pain Therapy^p^	—	—	—	—	—	✓	—	—	✓	—	—	—	—	—	2
Count across apps	2	1	7	1	16	13	0	9	11	3	7	1	3	0	—

^a^PE: pain education.

^b^AP: activity pacing.

^c^TB: thoughts and behavioral management.

^d^E: exercises (biomechanical/aerobic).

^e^R/B: relaxation/Breathing.

^f^M/M: meditation/Mindfulness.

^g^D: distraction techniques.

^h^ST: self-tailoring.

^j^SM: self-monitoring of symptoms.

^j^GS: goal setting and planning.

^k^PS: problem solving.

^l^PV: partnership between views of patient and health professionals.

^m^SS: social support.

^n^CR: cultural relevance.

^o^Criteria not met.

^p^Free app.

**Table 4:** Mean Mobile App Rating Scale scores of included apps.

App Name	MARS^a^ Categories					
	Engagement Section A	Functionality Section B	Aesthetics Section C	Information Section D	Subjective Quality Section E	App Quality Mean Score^b^ (A+B+C+D)
Aware—Meditation and Mindfulness	3.60	4.25	4.67	4.20	4.50	4.18
Counting Down to Relaxation	1.80	4.25	2.00	2.83	2.00	2.72
Curable: Back Pain, Migraine and Chronic Pain Relief	4.00	5.00	4.66	4.50	4.75	4.54
Freedom From Pain	2.60	4.00	2.67	3.50	1.75	3.19
General Pain Management Guided Imagery	3.00	4.75	2.33	3.50	2.00	3.40
Headspace: Guided Meditation	4.20	4.50	5.00	4.20	5.00	4.48
Meditations for Pain Relief	3.40	5.00	4.33	3.25	3.00	4.00
Mindfulness Coach^c^	2.80	4.75	4.00	4.33	2.75	3.97
Mindfulness Daily^c^	4.20	4.50	5.00	4.14	4.75	4.46
Mindfulness Meditation for Pain Relief-Jon Kabat-Zinn	2.60	3.50	3.00	3.00	1.50	3.03
Pain Control Hypnosis by Glenn Harrold	3.20	5.00	4.00	4.00	3.00	4.05
Pain Relief and Healing Meditation	2.60	4.00	3.00	2.67	1.75	3.07
Pain Relief Hypnosis PRO	3.80	4.75	4.67	3.67	3.25	4.22
PainScale-Pain Diary and Coach^c^	4.20	4.50	4.67	4.50	4.00	4.47
Relax—Stress and Anxiety Relief	3.60	4.75	5.00	3.67	4.00	4.25
Release Pain with Andrew Johnson	3.00	5.00	4.33	3.67	2.75	4.00
Sahaja Kundalini Meditation	2.20	4.50	3.33	3.00	2.00	3.26
SuperBetter^c^	4.00	4.00	4.33	4.33	4.25	4.17
Yoga For Pain Therapy^c^	2.20	4.50	3.33	2.33	1.25	3.09

^a^MARS: Mobile Apps Rating Scale.

^b^App quality mean scores were calculated based on mean scores from engagement, functionality, aesthetics and information quality.

^c^Free app.

## Discussion

### Principal Findings

Although this app review identified 19 apps that purported to support self-management of persistent pain, the comprehensiveness of app contents fostering ongoing self-management skills and functions was limited. Of interest, 2 of the 3 apps (PainScale-Pain Diary and Coach and SuperBetter) including the largest number of items to support self-management of pain were both free, suggesting the potential for using apps as a scalable, wide-reaching intervention to complement face-to-face care. None of the included apps provided information tailored to cultural needs of the user, and none had been evaluated in people with pain. Furthermore, features facilitating individual goal setting, peer support, and communicating health information with clinicians were infrequent.

A majority of the included apps (n=11) focused on delivering a single self-management strategy (eg, meditation and guided relaxation) for symptom management but lacked the features to facilitate core self-management skills (eg, goal setting and problem solving). Apps with more features facilitating core self-management skills have the potential for facilitating behavioral change [40], as identified in app-based systematic reviews promoting physical activity [41,42]. However, the self-management skills that are most influential in facilitating long-term behavior change in this clinical population remain unknown. A recent metasynthesis of qualitative studies identified that people with persistent pain valued ongoing support to sustain motivation to incorporate learnt self-management strategies after completing face-to-face treatments [11]. Overall, 3 of the included apps (Curable, Mindfulness Daily, and SuperBetter) did include 5 out of the 6 core self-management skills, suggesting that apps used after or alongside face-to-face care could be a feasible mechanism for providing ongoing self-management support.

Self-management strategies delivered for symptom management were predominantly mindfulness-based cognitive interventions. Mindfulness-based interventions such as stress reduction and cognitive therapy have been shown to improve long-term health outcomes in people with persistent pain [43]. Mindfulness-based apps had sophisticated features to facilitate thought and behavioral management. For example, apps featured weekly modules for the user to complete and had the capacity to self-monitor meditations via logs and setting reminders. By contrast, app facilitating guided relaxation in the form of guided imagery and hypnosis had only audio tracks with limited opportunities for tailoring to user needs. Inclusion of persuasive system design principles by means of task breakdown, providing reminders, and praise for achieving intended behavior and providing peer support with similar users has the potential to be persuasive to facilitate adherence to self-management strategies [44].

Pain education and activity pacing, 2 widely used self-management strategies were infrequently present in the included apps. For the purpose of the review, pain education was defined relative to concepts of pain neuroscience education, information on pain-stress-depression, medication, and sleep management. Pain neuroscience education has been shown to improve long-term improvements in pain-related disability and functioning [45]. Except for the Curable app, which provided comprehensive pain education via an interactive virtual pain coach, it is a significant omission that pain education and support for activity pacing were absent from most apps. For people with pain to successfully pace daily activities, preplanning, establishing user-centered activity goals, scheduling daily activities based on their symptoms, and prioritizing to achieve graded increment of activities are required [46]. These skills need to be supplemented by constant reinforcement, feedback, and monitoring [47]. Recent studies have shown the potential for wearable devices providing real-time feedback during daily activities and encouraging people to improve pacing [48,49]. Given the complexity in defining pacing, theoretical models guiding pacing intervention (operant theory and energy conservation) [46], and individualized nature of pacing [47], the potential to facilitate pacing via apps needs further investigation before widespread inclusion in apps.

Goal setting was found in only 3 of the included apps, a finding similar to previous app-reviews on persistent pain [24,31]. It has been shown to be an effective strategy to foster self-efficacy in people with pain [10]. Only 2 apps (Mindfulness Coach and Mindfulness Daily) used a SMART format (specific, measurable, achievable, realistic, and timed), whereas SuperBetter used gamified terms such as *quests*, *power ups*, and *bad guys* to foster goal setting. Despite the differences, further research is required to identify best-practice delivery of the goal-setting features in pain apps that would address the needs of users [31].

Social support can foster self-management [50], but it was featured in only 3 included apps. Although most of the apps allowed users to post activities to social media, which could be considered a means of facilitating peer support, this criterion was defined as providing access to peers with a similar health condition by providing informational, appraisal, and emotional support from their common disease experiences [51]. An app meeting this criterion would provide peer support via patients’ stories of living well, with pain or discussion forums embedded in the app. Online support communities to connect and share information have been shown to improve pain-related outcomes such as improved self-efficacy and self-reported global health [52,53]. Although issues related to confidentiality and privacy need to be considered before incorporation of these features into apps, people with persistent pain value the importance of peer support and peer validation as an important skill for long-term self-management [11].

None of the apps provided culturally tailored information, a finding similar to our previous review on pain self-management websites [54]. Although meeting all these cultural aspects in an app may not be possible and would be country or region specific, there is a need for providing information tailored to cultural beliefs, considering the existing health disparities across cultures (eg, ethnic and racial minorities) in pain prevalence and access to services [55]. From the New Zealand context, providing culturally appropriate information is important to meet the needs of New Zealand Māori—Indigenous people of New Zealand—who are at high risk of reporting persistent pain [56] and experience greater health inequities than any other ethnic group in New Zealand [13,57]. It is interesting to note that none of the previous app reviews [23,24,30,31,58] have investigated the provision of culturally tailored information despite the evidence to support that cultural beliefs and practices affect individual pain experiences [59] and adherence to active self-management strategies [27]. Currently, there is no guideline available for cultural tailoring of apps. Codesigning resources with specific cultural groups by involving significant others (eg, family members), using visual aids such as pictures, cartoons, and videos, and simplified text with less jargon have been shown to positively influence health beliefs and self-management strategies [60,61], suggesting the potential for apps as a medium for delivering culturally relevant information.

The included apps had high rating on the MARS. Apps in general scored highly in esthetics and often earned higher scores in engagement, resulting in apps that are easy to use, interactive, and promoted repeat use. Although 2 of our included apps (Headspace and SuperBetter) scored on the MARS item 19 for *evidence base* for improved health outcomes in people with anxiety and depression [38,39], no apps were validated in people with persistent pain, a finding similar to a previous app review on persistent back pain [30]. Furthermore, the mean MARS total score (3.82) for the included apps was higher than in the previous pain app review (2.36) [30]. Differences could be because of the large number of apps reviewed (n=61) compared with the present review (n=19).

The majority of included apps required payment (n=14,) ranging from a single payment of NZD $2.99 to NZD $25.99 per month, raising the question if pain apps can be an equitable intervention. This is particularly important when considering existing inequities among people with persistent pain. As those in lower socioeconomic areas are overrepresented in this clinical group [56], they are also therefore disproportionately impacted by financial barriers to accessing self-management interventions [62,63]. Interestingly, the 2 of the 3 highest scored apps from our self-management support evaluation and MARS rating (PainScale-Pain Diary and Coach and SuperBetter) were both free, suggesting that providing high-quality self-management support via apps can be feasible and equitable.

### Limitations

Although this is the first review to comprehensively assess the app contents using best-practice items to support self-management skills, including cultural tailoring in people with persistent pain, the following limitations need to be acknowledged in interpreting the results from this review. First, during our app screening process, we identified a number of popular apps for symptom monitoring (eg, Manage My Pain and My Pain Diary), which failed to meet our inclusion criteria because the present review had an inclusion criterion that required apps to provide information on pain management strategies. Although pain intensity assessment is an important aspect of overall self-management, pain intensity alone is a poor indicator of suffering and disability associated with persistent pain [64]. Furthermore, multidisciplinary cognitive behavioral therapy–based interventions focus on improving pain-related function instead of pain intensity [65]. Therefore, a comprehensive evaluation of symptom monitoring apps is beyond the scope of this review and would require another systematic review of apps. Although we were able to identify 2 apps (Brigham and Women's Hospital (BWH) Pain app and WebMD Pain Coach) from previous studies [31,66], these apps were not available at the New Zealand App Store and Google Play Store. Two popular apps (Pain Tricks and Pain Squad) were not included as they focused on children with persistent pain.

Secondly, our customized SMS-14 checklist, although based on evidence-based recommendations, has not been tested for validity. However, evidence suggests that technological interventions with more features facilitating core self-management skills (self-efficacy building, self-monitoring, goal setting, and problem solving) and support functions (peer support and cultural appropriateness) have the potential to foster long-term adaptive behavioral change [40-42]. Therefore, clinicians can be reasonably confident in the comprehensiveness of features facilitating self-management support from the apps included in the review. We acknowledge this is an area for further exploration as the most effective component of the multidisciplinary behavioral interventions remains unknown [5]. Next, only apps available in the New Zealand stores were included. This could have missed comprehensive pain self-management apps that are currently available in other countries (eg, BWH Pain app and Pain Toolkit). Finally, only app descriptions were used in the first screening stage, a standard practice in app-based reviews. Therefore, apps containing suitable self-management skills may have been missed if these skills were not described in the app store description.

### Conclusions

This app review evaluated the contents of apps fostering self-management support for people with persistent pain. Of the 3 apps (Curable, PainScale-Pain Diary and Coach and SuperBetter) that met the largest number of items to support skills in self-management of pain, 2 apps (PainScale-Pain Diary and Coach and SuperBetter) were free, suggesting the potential for using apps as a scalable, wide-reaching intervention to complement face-to-face care. However, none provided culturally tailored information. Although 2 apps (Headspace and SuperBetter) were validated to show improved health outcomes, none were tested in people with persistent pain. Both users and clinicians have to be aware of such limitations and make informed choices in using and recommending apps as a self-management tool. Concerted efforts are required among app developers, clinicians, and people with persistent pain in developing and testing apps for better integration of such technologies in clinical practice.

## Abbreviations

BWH: Brigham and Women's Hospital
ICC: intraclass correlation coefficient
ICD: International Classification of Diseases
MARS: Mobile App Rating Scale
SMS-14: 14-item self-management support checklist
